# Providence nighttime brace is as effective as fulltime Boston brace for female patients with adolescent idiopathic scoliosis: A retrospective analysis of a randomized cohort

**DOI:** 10.1016/j.xnsj.2022.100178

**Published:** 2022-10-22

**Authors:** Vojtech Capek, Olof Westin, Helena Brisby, Per Wessberg

**Affiliations:** a Department of Orthopedics, Institute of Clinical Sciences, Sahlgrenska Academy, University of Gothenburg, Goteborgsvagen 31, SE-431 80 Molndal, Sweden; b Spine Surgery Unit, Orthopedic Clinic, Sahlgrenska University Hospital, Bruna straket 11B, SE-413 45 Gothenburg, Sweden

## Abstract

**Background:**

Progressive moderate scoliotic curves in patients with adolescent idiopathic scoliosis (AIS) are usually treated with a fulltime brace, e.g., the Boston brace (BB). The Providence nighttime brace (PNB), is an alternative which is designed to reach the same treatment effectiveness by nighttime wear only. Few studies compared treatment effectiveness between full and nighttime bracing with contradictory results.

**Methods:**

Immature female patients older than 10 years with progressive moderate AIS curves with an apex below T6 were randomized into PNB (*n*=62) or BB (*n*=49) treatment. Inclusion criteria were AIS, age ≥ 10 years, no previous treatment, main curve Cobb angle 20°-40° and skeletal immaturity. The increase of the main curve by > 5° of Cobb angle at the final follow-up was established as the primary outcome measure. Secondary outcome measures included (1) the Scoliosis Research Society assessment criteria of effectiveness for brace studies, (2) progression of secondary curves, (3) in-brace correction and (4) compliance to the treatment. The patients were followed until 1 year after reaching maturity.

**Results:**

A total of 105 patients (*n*=62 and *n*=43 in PNB and BB group, respectively) completed the follow-up (95%). In the PNB group, 71% patients were treated successfully compared to 65% patients in the BB group (*p*=.67). No significant difference of the curve progression was found between the groups (3.1°±6.3° and 2.6°±8.3° in PNB and BB group, respectively; *p*=.73). No significant differences were found for the thoracic or thoracolumbar/lumbar subgroups. PNB showed a superior in-brace correction for all curve types. One of four secondary curves progressed > 5°. The compliance to the treatment was significantly higher in the PNB than BB group.

**Conclusions:**

Both brace regimes are equally effective in treating moderate AIS curves with apex of the main curve below T6 in immature female patients older than 10 years.

## Introduction

Adolescent idiopathic scoliosis (AIS) is a condition, in which a growing spine changes its form in the frontal, sagittal, and axial planes and develops a typical “S”- shaped deformity. It most commonly affects otherwise healthy adolescent females shortly after initiation of a rapid growth spurt period. Traditionally, milder forms of AIS (25°- 40° of Cobb angle) are treated with bracing [1]. The goal of such treatment is to limit the deformity during the growth spurt and prevent the development of more severe curves, that might require a surgical correction. Nowadays, there is no doubt that correctly indicated bracing has a positive effect on the curve progression [1,2]. The widely used fulltime brace, e.g., Boston brace (BB) is recommended to be worn 23 h per day, which affects the daily activities of a young patient and jeopardizes the compliance of the treatment [3], [4], [5]. Therefore, braces suitable for limited nighttime wear have been developed. The Charleston brace and Providence nighttime brace (PNB) belong to the most common types and their corrective mechanism is explained by applying hypercorrective forces on the scoliotic curve [6].

Several studies have shown that nighttime bracing can alter the natural course of the AIS in rapidly growing adolescents and thus prevent severe deformity from developing [6], [7], [8], [9], [10], [11]. However, studies comparing nighttime bracing with fulltime bracing have shown contradictory results, thereby not providing data allowing a general recommendation for nighttime bracing [12], [13], [14], [15]. Moreover, some authors suggest that the nighttime brace is more suitable for the treatment of thoracolumbar or lumbar (TL/L) curves rather than thoracic curves [6,13]. Finally, a deterioration of minor secondary curves due to hypercorrective forces of the nighttime brace has been described [16]. These retrospective studies are prone to selection bias and no comparative longitudinal fully prospective studies have been conducted.

Here, we present the first study of a prospectively followed cohort with patients randomized to treatment with fulltime or nighttime bracing conducted in immature female patients over 10 years with moderate AIS curves. The increase of the Cobb angle by > 5° at the last follow-up was the primary outcome measure. In addition, the Scoliosis Research Society (SRS) assessment criteria of effectiveness for brace studies, the compliance to the treatment and the course of the secondary/compensatory curves were assessed retrospectively.

## Materials and methods

### Study design

A retrospective cohort study on 111 consecutive AIS female patients with prospectively collected data in a clinical setting was conducted and approved by The Swedish Ethical Review Authority (DN: 2020-04364). Patients were recruited between 2004 and 2009 within a single institution specialized in both the bracing and surgical management of AIS. Since the SRS assessment criteria of effectiveness for brace studies were not available at the time of data collection (i.e., Risser ≤ 2, less than 1 year postmenarchal) [17], the inclusion criteria were chosen based on clinical practice at the time for inclusion at the institution. Only immature females with AIS over 10 years of age, Cobb angle of the main curve ranging 20° - 40°, apex at the level or below Th7 and/or with progression of the Cobb angle by > 5° during the last 6 months were included. Immaturity was defined as less than 1 year after menarche, Risser sign ≤ 2 or skeletal age of the hand ≤ 14 years according to Greulich and Pyle standards [18]. Exclusion criteria were non-idiopathic scoliosis, any neurologic abnormality at clinical examination and/or previous treatment for scoliosis. All consecutive patients, who met the criteria and consented to participate, were randomized to either of the two brace groups, i.e., girls born on even dates were assigned to the PNB group and those born on uneven dates were assigned to the BB group.

### Treatment procedure

**Fig. 1:**
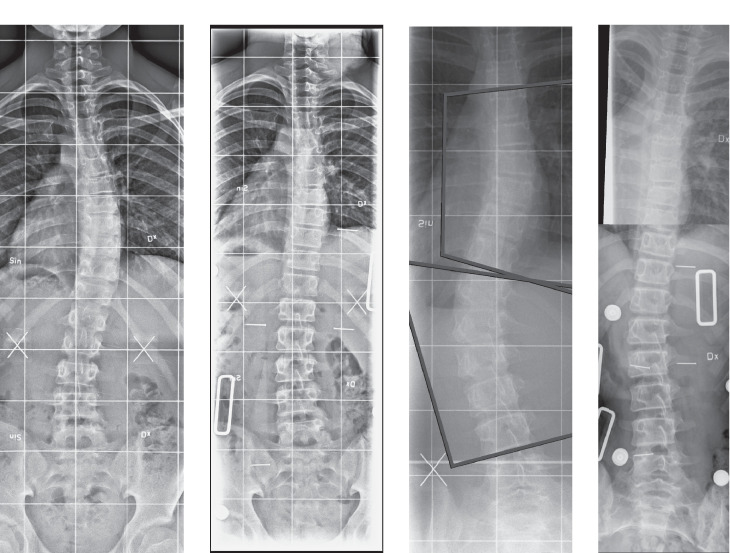
Comparison of the AIS curves. Left: Boston brace treatment of the thoracic AIS curve, pre-brace (28°) and in-brace standing (12°). Right: Providence brace treatment of the double AIS curve, pre-brace (36° thoracic and 32° lumbar curve) and in-brace supine (3° thoracic and 13° lumbar curve).

The PNB´s manufacturing, measurement and technique is based on applying over-corrective forces on the scoliotic curve and has been previously described elsewhere [6]. The fulltime brace (BB) was considered the gold standard treatment with a recommendation of wearing the brace 23 h/day followed by a 6 months period of weaning. After fitting the brace, a supine in-brace frontal radiograph (PNB) or standing in-brace frontal radiograph (BB) of the whole spine was obtained and Cobb angle measurements were noted (Fig. 1). Brace adjustments were then made to ensure maximal correction of the curves. New in-brace radiographs were then taken. All patients were followed by a specialized physiotherapist and orthopedic engineer for further adjustments until the optimal brace form was reached, usually this was achieved within 4–6 weeks after fitting the brace the first time. Clinical and radiological follow-ups with a standing frontal radiograph of the spine after a 24 h brace-free interval was taken every 6–12 months.

The compliance to the treatment was assessed by the clinicians and/or the physiotherapists and noted in the medical record at each visit. The patients with a low compliance were offered to change treatment group. Decisions to end treatment were identical for both treatments and based on skeletal maturity assessment (Risser sign ≥ 4 and/or skeletal age > 15 years according to Greulich and Pyle standards) or more than two years after menarche. All individuals were followed for at least one more visit 1 year after treatment cessation.

### Outcome variables

The Cobb angle was determined as the primary outcome variable of brace effectiveness and treatment success was defined as a progression of the main curve ≤ 5° between pre-brace and last follow-up radiographs. Lenke classification was used to describe different curve subtypes but for the research purposes, the patients were pooled into three main subgroups, i.e., main thoracic, main TL/L and double major curves. Both Lenke classification and all Cobb angle measurements were reviewed independently by two senior spine surgeons at pre-brace, in-brace and for all follow-up time points and intraclass correlation coefficient (ICC) was calculated. The mean of the two measurements of the same image was calculated if the inter-observer difference did not reach a cut-off value of 5°. Otherwise, the image was assessed by both reviewers together and a consensus was reached. The same process was used for disagreement on the Lenke classification.

The SRS assessment criteria of effectiveness for brace studies, i.e., progression of the main curve by > 5°, progression beyond > 45° or surgery, and progression of secondary curves and certain main curve types were analyzed as secondary outcomes. To be classified as failure due to surgery, the decision for surgery had to be made within 2 years after brace treatment cessation. The In-brace Correction (IBC) was defined as a difference between the pre-brace and in-brace Cobb angle and its correlation to the compliance was calculated.

The radiologic skeletal maturity was assessed by using Sanders staging (SS) of the hand radiographs and/or Risser sign (RS) of the iliac crest apophysis. There was a tendency to use RS at the beginning of the patients inclusions whereas a hand radiograph was more frequently used towards the end of the inclusion period. Therefore, the RS was transposed to SS system using the SS – RS matching proposed by Sanders in his previous work, i.e., SS 4 ≈ RS 0 and triradiate cartilage open; SS 5 ≈ RS 0 and triradiate cartilage closed; SS 6 ≈ RS 1-3; SS 7 ≈ RS 4; SS 8 ≈ RS 5[19]. Thus, the “Modified Sanders stage” variable is composed by a true SS, where available, and a RS matched to SS.

The compliance to the treatment is an ordinal categorical variable where the patients were retrospectively divided into four groups, i.e., compliance >75%, 50–75%, 25–49% and less than 25% of the prescribed brace time. The grouping was based on the retrospective review of the medical records recorded by the physician or the physiotherapist at each visit. The patient with an excellent adherence to the treatment or the minor compliance issues during less than 25% of the prescribed time were assigned to the group “compliance >75%”. The patients who had minor compliance issues between 25% and 50% of the prescribed time were assigned to the group “compliance 50–75%”, e.g., patients who were not able to wear the BB at school or a PNB patient who involuntarily “opened up” the brace some but not all the nights per week. The patients who had major compliance issues, e.g., PNB patients who were not able to were the brace more than 3 nights/week or the BB patients who were able to use the brace during nights only, were assigned to the group “compliance 25–49%”. All the patients who quit bracing after less than 25% of the prescribed period or used the brace less than 25% of the daily recommendation were assigned to the group “compliance <25%”. The compliance between the two treatments was compared.

### Statistical analysis

The power analysis was calculated based on previous studies considering the increase of the radiological Cobb angle > 5° being clinically important [7,20]. It was estimated that a minimum of 44 patients in each group was needed to detect a clinical important difference between the groups with a power of 80% and alpha level at 0.05. Allowing 10% loss to follow-up, 96 patients were required to complete the study. The within-groups results are presented as mean (standard deviation) and median (min; max) or counts (proportions). Continuous data such as Cobb angles and its change over time were compared using Fischer´s non-parametric premutation test since the data were not normally distributed. Also, Analysis of covariance (ANCOVA) with adjustment for initial curve magnitude, level of compliance and menarche status was used for primary outcome measure comparison. These potential confounders were chosen based on previous studies [17,21]. Fischer´s exact test was used for comparison of dichotomous variables. Mantel-Haenszel Chi square test and Chi square test were used for ordered and non-ordered categorical data, respectively. The Spearman correlation was used to calculate the relationship between the compliance and the IBC. The confidence intervals for dichotomous variables are presented as the unconditional exact confidence limits. If no exact limits could be computed, the asymptotic Wald confidence limits with continuity correction were calculated instead.

The two groups were compared using statistical tests described above and the main outcomes are presented as mean difference with 95% confidence intervals. The effect sizes were calculated. The subjects who crossed over to the other group changed the group early after the recruitment and thus were included in *as treated* analysis. All significance tests were two-sided and conducted at the 5% significance level. The statistical analysis was performed using SPSS v 26.0 (SPSS Inc., Chicago, IL, USA).

## Results

### Study population

A total of 111 immature female patients (>10 years of age) with AIS were included in the PNB (*n*= 62) and BB (*n*=49) groups. Age, curve characteristics, skeletal maturity, menarche status and length of the follow-up were similar between the groups (Table 1). Three patients had initial curves slightly outside of the primary inclusion criteria at the re-measurement of the radiographs (1 BB 52°, 1 BB 19° and 1 PNB 42°) and were included in the study. All included patients started brace treatment and underwent in-brace Cobb angle measurements.

**Table 1:** Baseline characteristics.

	Total (*n*=111)	Providence (*n*=62)	Boston (*n*=49)	p	Mean Δ (95% CI)
Age at bracing (y)	13.5 (1.2) 13.6 (10.9; 15.9) n=111	13.6 (1.1) 13.7 (10.9; 15.7) n=62	13.5 (1.2) 13.6 (11.0; 15.9) n=49	.84	0.05 (−0.40, 0.49)
Pre-brace main curve Cobb angle (°)	31.1 (5.3) 31 (19; 52) *n*=111	30.8 (5.0) 31 (20; 42) *n*=62	31.6 (5.7) 31 (19.5; 52) *n*=49	.47	−0.75 (−2.77, 1.28)
Pre-brace secondary curve Cobb angle (°)	20.6 (6.8) 20 (7; 36) *n*=111	20.5 (6.3) 20 (7; 36) *n*=62	20.7 (7.5) 20 (8; 35) *n*=49	.86	−0.23 (−2.85, 2.37)
Follow-up since brace cessation (y)	1.25 (0.57) 1.09 (0.4; 4.11) *n*=99*	1.27 (0.56) 1.09 (0.5; 4.1) *n*=58	1.23 (0.59) 1.05 (0.4; 3.2) *n*=41	.75	−0.04 (−0.27, 0.19)
Non-compliant		2 (3%)	5 (11%)	.14	−0.07 (−0.16, 0.29)
Modified Sanders stage					
2	3 (3.2%)	1 (1.9%)	2 (5.0%)		
3	14 (15.1%)	6 (11.3%)	8 (20.0%)		
4	25 (26.9%)	14 (26.4%)	11 (27.5%)		
5	13 (14.0%)	8 (15.1%)	5 (12.5%)		
6	30 (32.3%)	17 (32.1%)	13 (32.5%)		
7	8 (8.6%)	7 (13.2%)	1 (2.5%)	.084	
Type of the main curve					
Thoracic	63 (56.8%)	36 (58.1%)	27 (55.1%)		
TL/L	38 (34.2%)	22 (35.5%)	16 (32.7%)		
Double	10 (9.0%)	4 (6.5%)	6 (12.2%)	.57	
Menarche status					
Pre	57 (51.4%)	28 (43.8%)	29 (61.7%)		
Post	54 (48.6%)	36 (56.3%)	18 (38.3%)	.084	

For categorical variables n (%) is presented. For continuous variables Mean (SD) / Median (Min; Max) / n= is presented. For comparison between groups Fisher´s Exact test was used for dichotomous variables, Mantel-Haenszel Chi Square test was used for ordered categorical variables, Chi Square test was used for non-ordered categorical variables and the Fisher´s non-parametric permutation test was used for continuous

variables. The confidence interval for dichotomous variables is the unconditional exact confidence limits. If no exact limits can be

computed the asymptotic Wald confidence limits with continuity correction are calculated instead. The confidence interval for then mean difference between groups is based on Fishers non-parametric permutation test. Δ= difference between groups; y= years;TL/L=thoracolumbar/lumbar; *The patients who underwent surgery during brace treatment (n=6) and drop-outs (n=6) were excluded.

**Fig. 2:**
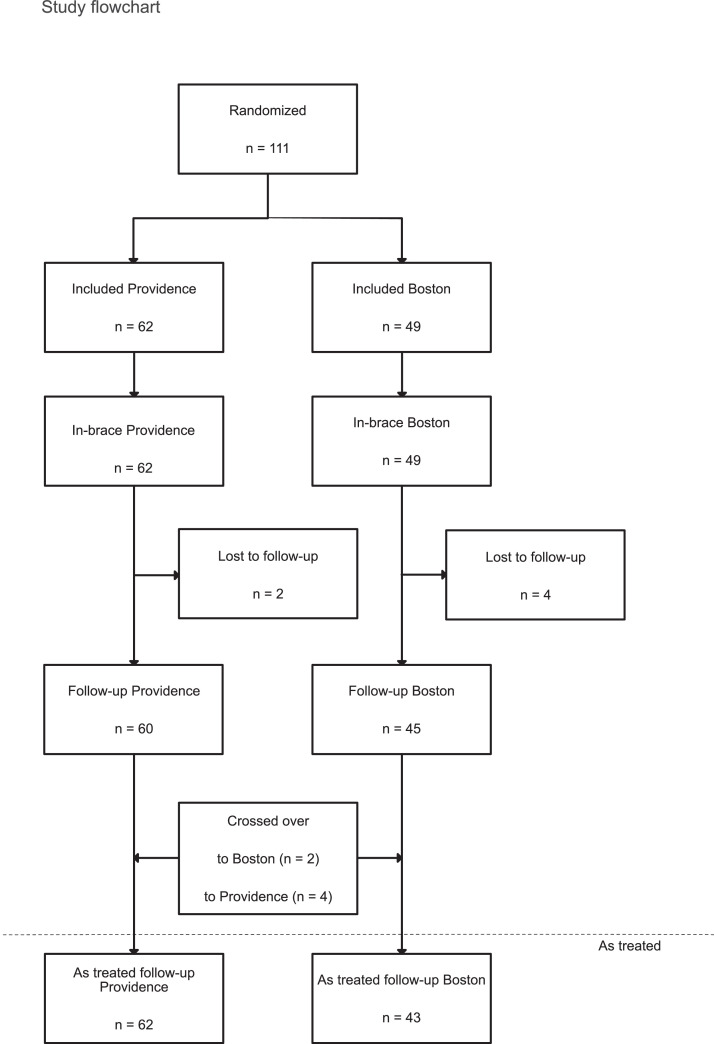
Study enrollment and treatment. All included patients were also available for in-brace measurements. Six patients were lost to follow-up and 60 and 45 patients in the Providence and Boston groups, respectively, finished the study. The patients were allowed to change the treatment group. Thus, 62 and 43 patients in the Providence and Boston groups, respectively, were included in the *as-treated* analysis.

At last follow-up, 105 subjects (95%) completed the study, two patients moved to another region and four missed the final follow-up for unknown reason. Two patients in the PNB and five in the BB group could not comply with the treatment and discontinued bracing before reaching maturity. These patients were included in the analysis. Two patients from the PNB and four patients from the BB group crossed over to the other group during the treatment and completed the follow-up (Fig. 2). The ICC of the Cobb angle measurements was 0.996 based on 626 radiographs. The mean follow-up was 15.2 and 14.8 months after treatment cessation in the PNB and BB group, respectively.

### Primary outcome – treatment success

No significant differences in the brace treatment success rate between the groups were found in the overall comparison or for the thoracic and TL/L curve subtypes. No conclusion could be drawn in the double major subgroup due to small counts (Table 2). Forty-four (71%) and 28 (65%) patients met the criteria for the treatment success in the PNB and BB groups, respectively, with a mean difference of 5.9% and 95% CI [−14.3, 26] (*p*=.67). In the PNB group, 64% of the thoracic curves, 78% of the TL/L curves and all double curves were treated successfully*.* Similar outcomes were found in the BB group (63% and 77% success rates, respectively). Of the six double curves in the BB group, 3 curves did not progress.

**Table 2:** Comparison of treatment success for Providence and Boston brace.

	Providence (*n*=62)	Boston (*n*=43)	p	Mean Δ (95% CI)
Success	44 (71.0%)	28 (65.1%)		5.9 (−14.3, 26.0)
Failure	18 (29.0%)	15 (34.9%)	.67	−5.9 (−26.0, 14.3)
Thoracic main curves	*n*=36	*n*=24		
Success	23 (63.9%)	15 (62.5%)		1.4 (−23.6, 27.0)
Failure	13 (36.1%)	9 (37.5%)	1.00	−1.4 (−27.0, 23.6)
TL/L main curves	*n*=23	*n*=13		
Success	18 (78.3%)	10 (76.9%)		1.3 (−26.5, 33.7)
Failure	5 (21.7%)	3 (23.1%)	1.00	−1.3 (−33.7, 26.5)
Double major curves*	*n*=3	*n*=6		
Success	3 (100)	3 (50)		
Failure	0 (0)	3 (50)		

Treatment failure was defined as increase of the Cobb angle by > 5° at last follow-up. For categorical variables n (%) is presented. For comparison between groups Fisher´s exact test (lowest 1-sided p value multiplied by 2) was used for dichotomous variables. The confidence interval for dichotomous variables was the unconditional exact

confidence limits. If no exact limits could be computed the asymptotic Wald confidence limits with continuity correction were calculated instead. Δ= difference between groups. * No statistical analysis was performed due to small counts. TL/L=thoracolumbar/lumbar

**Fig. 3:**
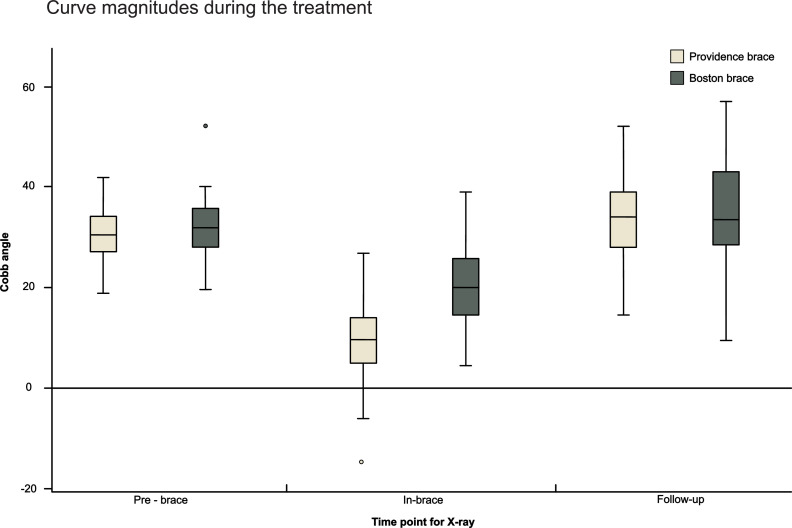
Clustered box plot of Cobb angle medians of main curves for the Boston and Providence braces at different time points. Upper and lower margins of the box delineate IQR (0.25–0.75). Whiskers show maximal and minimal values (1.5 IQR). The correction in Providence brace was significantly better than in the Boston brace (*p*<.0001). There was no statistically significant difference in effectiveness of the braces at final follow-up.

At the follow-up, the main curves reached 33.6° ± 8.3° and 34.6° ± 10.4° in the PNB and BB group on average, respectively (Table 3; Fig. 3). The adjusted mean difference of the main curve Cobb angles at the follow-up as well as the adjusted mean difference of the Cobb angle change between the PNB and BB groups was 2.0° with 95% CI [−0.9°, 4.9°] and was not statistically nor clinically significant (*p*=.17). Similar results were obtained for comparison of crude mean values (p=.57). The mean progression of the main curve from the initial Cobb angle measurement to the final follow-up was 3.1° ± 6.3° for the PNB and 2.6° ± 8.3° for the BB group (*p*= .73). The effect sizes for mean difference of both the final Cobb angles and progression of the curves between the groups were small. When analyzing the thoracic and TL/L curves separately, the thoracic curves progressed by 4.0° ± 6.7° and 1.8° ± 9.0° in the PNB group (*n*=36) and BB group (*n*=24), respectively (*p*=.29). The TL/L curves progressed by 1.8° ± 5.9° and 1.9° ± 6.7° in the PNB (*n*=23) and BB (*n*=13) group, respectively, with no difference between the groups (*p*=.99; Table 4).

**Table 3:** Comparison of Cobb angles at follow-up and its change during the brace treatment between Providence and Boston cohorts.

	Providence, (*n*=62)		Boston, (*n*=43)					
	Crude Mean (SD) Median (Min; Max)	Adjusted Mean* SEM (95% CI)	Crude Mean (SD) Median (Min; Max)	Adjusted Mean* SEM (95% CI)	p	Adjusted *p*-value*	Adjusted Means Δ (95% CI)	Effect size (unadjusted)
Follow-up main curve (°)	33.6 (8.3) 34 (14.5; 52)	34.9 0.9 (33.1, 36.6)	34.6 (10.4) 33.5 (9.5; 57)	32.8 1.1 (30.7, 35.0)	.57	.17	2.0 (−0.9, 4.9)	0.112
Cobb change main curve at follow-up (°)	3.1 (6.3) 2 (−14; 20)	3.7 0.9 (1.9, 5.5)	2.6 (8.3) 0 (−15.5; 19)	1.7 1.1 (−0.5, 3.8)	.73	.17	2.0 (−0.9, 4.9)	0.074
Follow-up secondary curve (°)	21.0 (7.4) 20.8 (5; 41)	21.5 0.8 (20.0, 23.1)	23.4 (9.7) 22 (6; 42)	22.7 0.9 (20.8, 24.6)	.16	.36	−1.2 (−3.6, 1.3)	0.281
Cobb change secondary curve at follow-up (°)	0.8 (6.1) 0.3 (−15; 15.5)	1.0 1.0 (−0.6, 2.5)	2.4 (6.2) 2 (−12; 22)	2.1 0.9 (0.3, 4.0)	.20	.36	−1.2 (−3.6, 1.3)	0.258

Cobb change is a difference between Cobb angle at final follow-up and pre-brace Cobb angle. For comparison between groups the Fisher´s non-parametric permutation test was used.

*) Adjusted for Pre-brace Cobb angle, Menarche status and Compliance *as treated* using Analysis of Covariance (ANCOVA). Δ = difference between groups; SEM = standard error of mean. Effect size is absolute difference in mean / pooled SD.

**Table 4:** Comparison of the change of Cobb angle at follow-up between the groups for thoracic and lumbar main curves.

	Providence	Boston	p	Mean Δ (95% CI)	Effect, Size
Cobb change of main **thoracic** curve at follow-up (°)	4.0 (6.7) 3 (−6; 20) *n*=36	1.8 (9.0) 1 (−15.5; 17) *n*=24	.29	2.2 (−1.9, 6.3)	0.29
Cobb change of main **TL/L** curve at follow-up (°)	1.8 (5.9) 2 (−14; 14) *n*=23	1.9 (6.7) 0 (−7; 15) *n*=13	.99	0.0 (−4.4, 4.4)	0.003

Mean (SD) / Median (Min; Max) / n are presented. For comparison between groups the Fisher´s non-parametric permutation

test was used. The confidence intervals for the mean differences between groups are based on Fisher´s non-parametric

permutation test. Effect size is absolute difference in means / pooled SD. Δ = difference between the groups. TL/L=thoracolumbar/lumbar

### Secondary outcomes

The overall outcome of the SRS treatment failure was identical with the results for one of its subdomains, i.e., reaching the threshold of > 5° curve progression, and hence identical with the primary outcome of this study. Consequently, all the curves that progressed beyond the threshold of 45° or were subjected to a surgery in the present study had also increased by > 5°. The threshold of > 45° for the main curve was reached by 5 (8%) and 7 (16%) patients in the PNB and BB group, respectively (*p*=.32). Surgical treatment was performed in nine (15%) and seven (16%) patients in each group, respectively (*p*= 1.00), of whom six patients (three patients in each group) were operated during the ongoing brace treatment due to a rapid curve progression (Table 5). The characteristics of the surgically treated patients are listed in Supplementary Table 1. An increase of the secondary curve > 5° was observed in 13 patients in each group (30% in BB and 21% in PNB groups; *p*= .28). Altogether, 75% of the secondary curves did not progress and none reached the 45° cut-off or was subjected to subsequent surgery. There was no difference in progression of the secondary curves between the groups (Table 3).

**Table 5:** Comparison of treatment failure according to SRS criteria for Providence and Boston brace.

	Providence, (*n*=62)	Boston, (*n*=43)	p	Mean Δ (95% CI)
Overall SRS failure main curve	18 (29.0%)	15 (34.9%)	.67	−5.9 (−26.0; 14.3)
Increase of main Cobb > 5°	18 (29.0%)	15 (34.9%)	.67	−5.9 (−26.0; 14.3)
Final Cobb main curve > 45°	5 (8.1%)	7 (16.3%)	.32	−8.2 (−23.1; 6.7)
Surgery	9 (14.5%)	7 (16.3%)	1.00	−1.8 (−17.8; 14.3)
Thoracic main curves	*n*=36	*n*=24		
Overall SRS failure main curve	13 (36.1%)	9 (37.5%)	1.00	−1.4 (−27.0; 23.6)
Lumbar main curves	*n*=23	*n*=13		
Overall SRS failure main curve	5 (21.7%)	3 (23.1%)	1.00	−1.3 (−33.7; 26.5)
Double curves	*n*=6	*n*=3		
Overall SRS failure main curve*	3 (50)	0 (0)		

For categorical variables n (%) is presented. For comparison between groups Fisher´s exact test (lowest 1-sided p value multiplied by 2) was used for dichotomous variables. The confidence interval for dichotomous variables is the unconditional exact

confidence limits. If no exact limits can be computed the asymptotic Wald confidence limits with continuity correction are calculated instead. Δ= difference between groups. * No statistical analysis was performed due to small counts.

### In-brace correction

The PNB showed a superior IBC in all curve types compared to the BB (Table 6), although both braces showed a significant correction. The main curve regressed in brace by 70 % and 37.5 % in the PNB and BB, respectively (*p*< .001). The overall main curve IBC was −21.8° ± 6.5° and −11.2° ± 5.6° in the PNB and BB group, respectively, with a mean difference between the groups of 10.6° with 95% CI [8.3°, 12.9°] favoring the PNB (p<.0001). The amount of correction of the secondary curves was similar to the correction of the primary curves for both braces in favor of the PNB. The largest difference in IBC between the cohorts was observed in the main TL/L group where the PNB showed 12° better correction than BB (95% CI [7.8°, 16.2°]). The main thoracic curves showed inferior correction compared to the main TL/L curves in both groups. The effect sizes for mean difference between the brace groups were large for all outcomes.

**Table 6:** In-brace Cobb angles and brace correction of the curves in both cohorts.

	Providence, (*n*=62)	Boston, (*n*=49)	*p*	Mean Δ (95% CI)	Effect, Size
In-brace correction main curve (%)	70.0	37.5	**< .001**	32.5 (23.9;41.1)	1.44
In-brace main curve (°)	9.0 (7.1) 9.3 (−15; 22)	20.4 (8.2) 20.0 (4.5; 39)	**< .0001**	−11.4 (−14.2; −8.6)	1.50
In-brace secondary curve (°)	6.7 (5.3) 6.3 (−2; 21)	14.9 (6.5) 15.5 (2; 29.5)	**< .0001**	−8.3 (−10.5; −6.1)	1.41
Correction main curve (°)	−21.8 (6.5) −21.5 (−39; −8)	−11.2 (5.6) −12 (−25; −1)	**< .0001**	−10.6 (−12.9; −8.3)	1.74
Correction secondary curve (°)	−13.8 (7.5) −13.3 (−33; 4)	−5.8 (4.9) −6 (−15; 5)	**< .0001**	−8.1 (−10.5; −5.6)	1.25
**Thoracic curves only**	*n*=36	*n*=27			
In-brace correction main curve (%)	63.4	32.6	**< .001**	30.8 (20.3;41.3)	1.50
Correction main curve (°)	−19.4 (4.7) −20 (−28.5; −10)	−9.7 (6.0) −8.5 (−22; -1)	**< .0001**	−9.7 (−12.4; −7.0)	1.84
Correction secondary curve (°)	−14.5 (6.3) −14.8 (−25.5; 1)	−5.6 (4.8) −7 (−14; 4)	**< .0001**	−9.0 (−11.9; −6.1)	1.56
**Lumbar curves only**	*n*=22	*n*=16			
In-brace correction main curve (%)	80.3	47.2	**< .001**	33.1 (16.3;49.9)	1.34
Correction main curve (°)	−26.0 (7.4) −26.8 (−39; -8)	−14.0 (4.5) −14.3 (−25; −4)	**< .0001**	−12.0 (−16.2; −7.8)	1.89
Correction secondary curve (°)	−10.6 (6.8) −11.3 (−22; 4)	−4.03 (4.33) −3.5 (−12.5; 5)	**.001**	−6.5 (−10.4; −2.6)	1.10
**Double curves only**	*n*=4	*n*=6			
Correction main curve (°)	−20.1 (4.0) −18.8 (−26; −17)	−10.3 (3.7) −10.5 (−15.5; −4)	**0.01**	−9.9 (−15.5; −4.5)	2.59
Correction secondary curve (°)	−25.1 (9.1) −25.5 (−33; −16.5)	−11.3 (2.5) −11 (−15; −8)	**0.01**	−13.9 (−23.5; −4.3)	2.35

In-brace correction (%) is the proportion of correction to the initial curve. In-brace correction (°) is a difference between the initial Cobb angle and the in-brace Cobb angle. Negative values suggest regression of scoliosis in brace. Labels of the sub-groups reflect the main curve. Main curve of a double curve was always the largest one. Mean (SD) / Median (Min; Max). For comparison between groups the Fisher´s non-parametric permutation test was used. The confidence interval for mean difference between groups is based on the Fisher´s non-parametric permutation test. Effect size is absolute difference in mean / pooled SD. Δ= difference between groups.

### Compliance

The compliance to the treatment was significantly higher in the PNB group in which 76% patients were able to wear the brace more than 75% of the prescribed time compared to only 56% in the BB group (*p*= .017; Table 7). Moreover, 28% patients in the BB group were not able to were the brace more than 50% of the prescribed time compared to only 10% in the PNB group. The Spearman correlation showed no significant correlation between IBC and compliance (*r*= −0.06; *p*= .51).

**Table 7:** Compliance.

	Providence (*n*=62)	Boston (*n*=43)	*p*-value
Compliance As Treated			
>75%	47 (75.8%)	24 (55.8%)	
50–75%	9 (14.5%)	7 (16.3%)	
25–49%	4 (6.5%)	9 (20.9%)	
<25%	2 (3.2%)	3 (7.0%)	0.017

The patients were divided into four groups depending on the overall compliance to the treatment. For categorical variables n (%) is presented.

For comparison between groups the Mantel-Haenszel Chi Square test was used for ordered categorical variables.

## Discussion

This is, to our knowledge, the largest study comparing fulltime and nighttime brace treatments with prospectively followed randomized patients and a follow-up rate of 95%. The radiographs were reviewed by two independent observers with an excellent ICC. No significant differences in the success rates between the groups were found. The nighttime bracing (PNB) showed a similar effectiveness in treatment of the thoracic curves as the fulltime bracing (BB). Moreover, the PNB showed a superior in-brace correction of both thoracic and TL/L curves regardless of whether the curve was primary or secondary. The effectiveness of the PNB has mostly been investigated in cohort studies, of which only the most recent studies applied the SRS criteria for brace success [6,8,9,11] and three studies directly compared the effectiveness of BB and PNB brace treatments with varying results [12,13,22]. Also, a recently published meta-analysis concluded that nighttime bracing could be an alternative to fulltime bracing in patients with TL/L curves and Risser 1 or 2, but the recommendation was limited by the quality and the sample size of the included studies [14].

In the current study, the overall success rates of 71% and 65% for PNB and BB, respectively, are in line with some of the previous reports and are proven to be significantly better opposed to the natural course of the AIS [1,2]. Our results are comparable to the outcome reported by Yrjonen et al., who performed the first comparative study with 36 prospectively followed patients fitted with the PNB and 36 retrospectively elected matched individuals treated with the BB (success rate of 73% for PNB and 78% for BB) [13]. In a retrospective single-cohort study, D´Amato showed a comparable outcome of the PNB to fulltime brace treatment in historical cohorts, with an overall 74% success rate in the PNB group, where the TL/L curves showed by far the best outcome (93% success) [6]. On the contrary, Janicki et al., comparing PNB and BB treatments and rigorously following the SRS inclusion criteria, reported poor outcomes for both braces with success rates of 42% and 15%, respectively [12].

The compliance is a strong factor influencing the effectiveness of the brace treatment [21,23,24]. Nighttime braces are usually tolerated better compared to fulltime braces [3]. Compliance monitors, e.g., temperature loggers, have been previously used in several studies on fulltime bracing [2,23,25] and in one study on nighttime bracing [3]. Katz et al. found that a minimum of 12 h treatment per day in the fulltime brace (Boston) is needed to make the difference in the curve progression compared to the natural course. Although there are no such data available on nighttime bracing, it is reasonable to assume that the more the brace is being worn the better effectiveness it can reach. Regarding the studies on nighttime bracing, Bohl et al. categorized the patients with any comment on compliance as noncompliant. To our knowledge, there are no other studies on nighttime bracing that included the compliance as a parameter of brace effectiveness.

Our study is the first of the kind that compares compliance of the brace treatment in the consecutive patients´ cohort and two different treatments. The PNB shows significantly better compliance than the BB. Despite that, the overall unadjusted effectiveness of both treatments is comparable. Interestingly, the mean difference in the Cobb angle change throughout the treatment between the PNB and the BB groups increased after the adjustment for age, menarche status and the grade of compliance in favor of the BB 2.0° with 95% CI [−0.9°, 4.9°]. Therefore, the post-hoc analysis was performed to compare a subgroup of highly compliant patients only (compliance > 75%; *n*=47 in PNB and *n*=24 in BB group). In this comparison, the mean difference of the Cobb angle change increased to 2.6° with 95% CI [−0.9°; 6.2°] with a potential clinical importance for > 5°Cobb angle change during the treatment in favor of the BB, however, the statistical significance was not reached (*p*=.14). A more robust study would be necessary to investigate this further.

The effectiveness of the nighttime bracing on the course of thoracic curves has been questioned [6,26]. Recently, Ohrt-Nissen et al. found no significant differences in the success rates between PNB and BB treatments of the thoracic curves only, although, the success rates were low in both groups, 38% and 45% respectively [22]. Similarly, both brace treatments in our study exhibit inferior success rates for treatment of the thoracic curves compared to the lumbar curves with a statistically non-significant mean difference of the curve progression of 2.2° between the PNB and BB. However, the 95% CI [−1.9, 6.3] could point at a potential clinical importance in favor of the BB.

A negative effect of the hypercorrective forces during nighttime bracing on the secondary curves has been described. Price et al. reported the long-term results of the hypercorrective nighttime Charleston brace in a single cohort comprised of 98 patients [7]. Apart from the acceptable outcome in 79% of patients, the effect on the double curves was reported as poor and four patients underwent surgery due to a progression of the secondary curve. It was hypothesized that “unbending” of one curve might have deteriorated the opposite curve. Our data show that the progression of the secondary curves followed a similar pattern as seen for the primary curves with a success rate of 75% and no patients subjected to surgery or having reached the 45° threshold. This finding may be explained by different corrective mechanisms between the Providence brace and the Charleston brace. While the Charleston brace “unbends” the main curve in one direction, the overcorrection of the PNB is reached by strategically placed pressure pads in the brace enabling a correction of the two opposite curves.

The IBC rate is one of the predictors of successful treatment using conventional fulltime bracing [27,28]. As for the PNB, several studies have demonstrated a similar relationship [6,13,29]. The IBC rates for PNB vary among the studies (68% - 111%) and thoracic and double curves generally demonstrate lower rates of correction than TL/L curves. Compared to the BB cohort, the current study demonstrates twice as good IBC for all the curves in the PNB group, thoracic and double curves included. The effect sizes for mean differences between the groups for the overall IBC as well as the outcomes of the subgroups were large (1.1–2.6) suggesting a large effect of the correction in the PNB compared to the BB. This difference can be considered as the basis for the good effect of the PNB in spite of the short time of daily ware.

### Limitations

The present study has several limitations. The skeletal maturity assessment changed from iliac crest radiographs to hand radiographs during the course of the study. Currently, since we find the SS more useful, we attempted to match the RS to SS system according to the approximation described by Sanders [19]. Furthermore, no compliance monitoring was available at our clinics at the time of the study. Instead, we attempted to retrospectively stratify the patients into four groups based on the comments on adherence to the treatment in the medical records. Although we believe that the quality of the information was sufficient to justify this approach, the underreporting of compliance issues could might have occurred as shown in previous studies [30]. On the other hand, this study compared two distinct devices with most likely different inherent potentials for compliance. The primary aim of the study was not to compare the compliance between the two treatments but to compare the overall effectiveness in the clinical settings with both more and less compliant patients equally distributed into the groups through randomization.

Considering a better compliance compared to a fulltime brace, the PNB has in later years become the treatment of choice for this patient population at our clinic. However, the study population is rather heterogenous with both more or less mature patients included. We did not perform any sub-analysis based on the state of maturity due to the limited power. Although, we share a similar experience as that recently published by Buyuk et al. in the systematic review that a nighttime brace (e.g., PNB) can be used safely at least for more mature patients (Risser > 0)[14]. We have not found any differences in the treatment effectiveness between the thoracic and TL/L curves and thus we apply the same treatment strategy for both curve types, also supported by the findings by Orth-Nissen et al[22]. Whether the PNB is a sufficient treatment for less mature AIS patients still needs to be clarified. In our institution, the BB is still the gold standard for the juvenile patients (triradiate cartilage open) or those with a poor compliance to the PNB.

## Conclusions

In conclusion, the PNB is as effective as the BB for treatment of all types of moderate (20°- 40°) AIS curves with apex at the level or below Th7 in growing skeletally immature females. The compliance to the treatment is better in the PNB treatment than in the BB treatment. The secondary curves do not deteriorate due to overcorrection in the Providence nighttime brace.

## Conflict of Interest

One or more of the authors declare financial or professional relationships on ICMJE-NASSJ disclosure forms.

## Appendix Supplementary materials

Image, application 1

## References

[1] Nachemson A.L., Peterson L.E. (1995). Effectiveness of treatment with a brace in girls who have adolescent idiopathic scoliosis. A prospective, controlled study based on data from the brace study of the scoliosis research society. J Bone Joint Surg Am.

[2] Weinstein S.L. (2013). Effects of bracing in adolescents with idiopathic scoliosis. N Engl J Med.

[3] Antoine L. (2020). Compliance with night-time overcorrection bracing in adolescent idiopathic scoliosis: result from a cohort follow-up. Med Eng Phys.

[4] Climent J.M., Sanchez J. (1999). Impact of the type of brace on the quality of life of adolescents with spine deformities. Spine (Phila Pa 1976).

[5] Rowe D.E. (1997). A meta-analysis of the efficacy of non-operative treatments for idiopathic scoliosis. J Bone Joint Surg Am.

[6] D'Amato C.R., Griggs S., McCoy B. (2001). Nighttime bracing with the Providence brace in adolescent girls with idiopathic scoliosis. Spine (Phila Pa 1976).

[7] Price C.T. (1997). Nighttime bracing for adolescent idiopathic scoliosis with the Charleston bending brace: long-term follow-up. J Pediatr Orthop.

[8] Simony A. (2019). Providence nighttime bracing is effective in treatment for adolescent idiopathic scoliosis even in curves larger than 35 degrees. Eur Spine J.

[9] Davis L. (2019). Nighttime bracing with the Providence thoracolumbosacral orthosis for treatment of adolescent idiopathic scoliosis: a retrospective consecutive clinical series. Prosthet Orthot Int.

[10] Gepstein R. (2002). Effectiveness of the Charleston bending brace in the treatment of single-curve idiopathic scoliosis. J Pediatr Orthop.

[11] Bohl D.D. (2014). Effectiveness of providence nighttime bracing in patients with adolescent idiopathic scoliosis. Orthopedics.

[12] Janicki J.A. (2007). A comparison of the thoracolumbosacral orthoses and providence orthosis in the treatment of adolescent idiopathic scoliosis: results using the new SRS inclusion and assessment criteria for bracing studies. J Pediatr Orthop.

[13] Yrjonen T. (2006). Effectiveness of the Providence nighttime bracing in adolescent idiopathic scoliosis: a comparative study of 36 female patients. Eur Spine J.

[14] Buyuk A.F. (2022). Is nighttime bracing effective in the treatment of adolescent idiopathic scoliosis? A meta-analysis and systematic review based on scoliosis research society guidelines. Spine Deform.

[15] Luaks K. (2021). Boston vs. Providence brace in treatment of adolescent idiopathic scoliosis. Stud Health Technol Inform.

[16] Price C.T. (1990). Nighttime bracing for adolescent idiopathic scoliosis with the Charleston bending brace. Preliminary report. Spine (Phila Pa 1976).

[17] Richards B.S. (2005). Standardization of criteria for adolescent idiopathic scoliosis brace studies: SRS committee on bracing and nonoperative management. Spine (Phila Pa 1976).

[18] Greulich, W.W. and S.I. Pyle, Radiographic atlas of skeletal development of the hand and the wrist : William Walter Greulich, S. Idell Pyle. 2. ed. 256 s.

[19] Sanders J.O. (2008). Predicting scoliosis progression from skeletal maturity: a simplified classification during adolescence. J Bone Joint Surg Am.

[20] Howard A., Wright J.G., Hedden D. (1998). A comparative study of TLSO, Charleston, and Milwaukee braces for idiopathic scoliosis. Spine (Phila Pa 1976).

[21] Hawary R.E. (2019). Brace treatment in adolescent idiopathic scoliosis: risk factors for failure-a literature review. Spine J.

[22] Ohrt-Nissen S. (2019). Conservative treatment of main thoracic adolescent idiopathic scoliosis: Full-time or nighttime bracing?. J Orthop Surg (Hong Kong.

[23] Katz D.E. (2010). Brace wear control of curve progression in adolescent idiopathic scoliosis. J Bone Joint Surg Am.

[24] Rahman T. (2005). The association between brace compliance and outcome for patients with idiopathic scoliosis. J Pediatr Orthop.

[25] Donzelli S. (2017). Adolescents with idiopathic scoliosis and their parents have a positive attitude towards the thermobrace monitor: results from a survey. Scoliosis Spinal Disord.

[26] Katz D.E. (1976). A comparison between the Boston brace and the Charleston bending brace in adolescent idiopathic scoliosis. Spine (Phila Pa.

[27] Katz D.E., Durrani A.A. (2001). Factors that influence outcome in bracing large curves in patients with adolescent idiopathic scoliosis. Spine (Phila Pa 1976).

[28] Xu L. (2017). Initial correction rate can be predictive of the outcome of brace treatment in patients with adolescent idiopathic scoliosis. Clin Spine Surg.

[29] Ohrt-Nissen S. (2016). Flexibility predicts curve progression in providence nighttime bracing of patients with adolescent idiopathic scoliosis. Spine (Phila Pa 1976).

[30] Takemitsu M. (2004). Compliance monitoring of brace treatment for patients with idiopathic scoliosis. Spine (Phila Pa 1976).

