# Supplementary material for: Providence nighttime brace is as effective as fulltime Boston brace for female patients with adolescent idiopathic scoliosis: A retrospective analysis of a randomized cohort
Source: N Am Spine Soc J. 2022 Oct 22;12:100178. doi: 10.1016/j.xnsj.2022.100178 (PMC9706154; doi:10.1016/j.xnsj.2022.100178)
Supplement: Supplementary file 1 [file mmc1.docx]

| Supplementary Table1  List of the patients, with selected variables, who underwent the surgical treatment during bracing or less than 2 years after the bracing´s cessation. | | | | | | | | | | |
| --- | --- | --- | --- | --- | --- | --- | --- | --- | --- | --- |
|  |  |  |  |  |  |  |  |  |  |  |
| Study nr. | Lenke | Brace type | Age | Menarche | Sanders | Main Cobb pre-brace | Main Cobb in-brace | Main Cobb final | Surgery in brace | Low compliance |
| 3 | 3C | 1 | 13.5 | 0 | N/A | 38 | 34 | 57 | 1 | 0 |
| 6 | 1C | 1 | 14.4 | 1 | 5 | 38 | 33 | 48 | 0 | 0 |
| 9 | 1B | 1 | 11.4 | 0 | 3 | 34 | 27 | 46 | 0 | 0 |
| 10 | 2A | 1 | 11.4 | 0 | 2 | 35 | 34 | 43 | 1 | 0 |
| 16 | 6C | 1 | 14.5 | N/A | 6 | 36 | 32 | 42 | 0 | 0 |
| 36 | 6C | 1 | 14.6 | 1 | 6 | 33 | 21 | 47 | 0 | 0 |
| 49 | 5C | 1 | 14.5 | 1 | 6 | 35 | 15 | 50 | 0 | 0 |
| 29 | 1B | 2 | 11.7 | 0 | 4 | 28 | 27 | 48 | 1 | 0 |
| 60 | 1B | 2 | 13.5 | 0 | 5 | 36 | 17 | 47 | 0 | 0 |
| 63 | 1A | 2 | 11.8 | 0 | 6 | 32 | 22 | 50 | 0 | 0 |
| 67 | 1B | 2 | 13.5 | 0 | 5 | 34 | 14 | 45 | 1 | 0 |
| 75 | 6C | 2 | 15.5 | 0 | 6 | 38 | 14 | 52 | 0 | 0 |
| 76 | 1B | 2 | 11.0 | 0 | 5 | 32 | 12 | 42 | 0 | 0 |
| 94 | 1B | 2 | 13.4 | 0 | N/A | 21 | 6 | 40 | 0 | 1 |
| 101 | 6C | 2 | 13.5 | 1 | N/A | 33 | -6 | 43 | 1 | 0 |
| 107 | 6C | 2 | 13.5 | 1 | 4 | 42 | 8 | 50 | 1 | 1 |
| Brace type - 1=Boston, 2=Providence. Age - age at bracing. Menarche – 0 = pre-menarche; 1 = post-menarche. Sanders = modified Sanders stage at bracing start. Main Cobb pre-brace = Cobb angle of the main curve at bracing start. Main Cobb in-brace = Cobb angle of the main curve in brace. Main Cobb final = Cobb angle of the main curve before surgery. Surgery in brace – 1 = surgery was performed during bracing; 0 = surgery was performed after bracing. Low compliance – 1 = bracing was discontinued before reaching maturity due to low compliance; 0 = the patient completed the treatment. | | | | | | | | | | |
|  |  |  |  |  |  |  |  |  |  |  |
|  |  |  |  |  |  |  |  |  |  |  |
|  |  |  |  |  |  |  |  |  |  |  |
